# Using Biofeedback while Immersed in a Stressful Videogame Increases the Effectiveness of Stress Management Skills in Soldiers

**DOI:** 10.1371/journal.pone.0036169

**Published:** 2012-04-27

**Authors:** Stéphane Bouchard, François Bernier, Éric Boivin, Brian Morin, Geneviève Robillard

**Affiliations:** 1 Université du Québec en Outaouais, Gatineau, Québec, Canada; 2 Laboratoire de Cyberpsychologie de l' Université du Québec en Outaouais, Gatineau, Québec, Canada; 3 Defence Research and Development Canada – ValCartier, Québec, Canada; 4 Clinique de Psychologie Cognitive, Québec, Canada; Universidad Europea de Madrid, Spain

## Abstract

This study assessed the efficacy of using visual and auditory biofeedback while immersed in a tridimensional videogame to practice a stress management skill (tactical breathing). All 41 participants were soldiers who had previously received basic stress management training and first aid training in combat. On the first day, they received a 15-minute refresher briefing and were randomly assigned to either: (a) no additional stress management training (SMT) for three days, or (b) 30-minute sessions (one per day for three days) of biofeedback-assisted SMT while immersed in a horror/first-person shooter game. The training was performed in a dark and enclosed environment using a 50-inch television with active stereoscopic display and loudspeakers. On the last day, all participants underwent a live simulated ambush with an improvised explosive device, where they had to provide first aid to a wounded soldier. Stress levels were measured with salivary cortisol collected when waking-up, before and after the live simulation. Stress was also measured with heart rate at baseline, during an apprehension phase, and during the live simulation. Repeated-measure ANOVAs and ANCOVAs confirmed that practicing SMT was effective in reducing stress. Results are discussed in terms of the advantages of the proposed program for military personnel and the need to practice SMT.

## Introduction

Mental resilience (i.e, the capacity to withstand, cope or recover from stress and adversity) [1]–[4] is now receiving significant attention among the military, as illustrated by an opening article for a special issue of *American Psychologist* by the U.S. Army Chief of Staff, General George W. Casey [5]. Related concepts have been discussed in the literature, such as mental readiness [6], mental toughness [7], or emotional resilience [8]. Soldiers are frequently exposed to traumatic events and acute stressors, which is associated with considerable rates of posttraumatic stress symptoms [9]. Research has consequently been increasingly focusing on the prevention of the negative effects of trauma exposure in soldiers. It is believed that fostering better resiliency in soldiers could not only positively improve their ability to cope with stress, but also reduces psychological wounds in the long run and improves their performance in theaters of operations.

Mental resilience draws on factors such as emotion regulation (i.e., the ability to regulate positive and negative emotions) [8] and, in a broader sense, self-regulation (i.e., “the ability to regulate impulses, thinking, emotions, and behaviors to achieve goals, as well as the willingness and ability to express emotions (p.27)” [3]. Building psychological strength before being deployed implies building psychological fitness and learning to manage emotions more positively, efficiently and with flexibility [5], [10]–[11].

Increasing resilience through the acquisition of self-regulation or, more narrowly, emotion regulation skills, are common objectives for psychological training programs aimed at military personnel. A few prevention programs have already been developed for soldiers and military personnel to build resilience and reduce psychological injuries: Battlemind Training [12] Road to Mental Readiness [13], and Programme d'Entraînement à la Resilience Militaire [14].

In Canada, the Programme d'Entraînement à la Résilience Militaire (PERM) has been implemented in the past and has now evolved into the Road to Mental Resilience program [13]. The general idea is to prevent psychological injuries related to operational stress and improve resilience both in theaters of operations and in garrison. It is composed of different modules that target soldiers, their superiors, spouses and life-partners and mental health professionals. It is based on a cognitive-behavioral/bio-psychosocial approach and includes numerous stress management training (SMT) strategies, namely goal setting, relaxation, diaphragmatic and controlled breathing, mental rehearsal and self-talk. The key learning objectives are understanding stress reactions, identifying challenges of deployment and their impact, learning and applying strategies to mitigate the impact of stress, and recognizing when and where to seek support.

In the U.S., one of the first largely implemented mental preparedness programs was Battlemind [12]. Developed by the Walter Reed Army Institute of Research, it aimed at mentally preparing soldiers to the rigors of combat and other military deployments in order to foster better mental health post-deployment. However, this program is now being replaced by a new one that integrates some aspects of Battlemind but focuses even more on resilience as a mean of prevention and with a stronger influence from positive psychology: the Comprehensive Soldier Fitness program [10].

The main goal of the Comprehensive Soldier Fitness is to enhance psychological resilience in members of the Army community. This program not only focuses on the prevention of psychological disorders in soldiers but also aims at enhancing psychological strengths already present in military personnel [5], [10]. More specifically, this program is being implemented with the goal of increasing psychological growth following combat exposure for an increased number of soldiers and of decreasing the number of soldiers who develop disorders related to stress [10]. This program promotes a holistic approach that focuses on five dimensions associated with resilience: physical, social, emotional, spiritual and familial [5]. It is delivered in four major modules: (a) online self-assessment that allows the identification of resilience strengths; (b) online self-help modules adapted to each soldier in regards to the results of the self-assessment (individualized training); (c) mandatory resilience training at the initial entry into the U.S. Army; (d) training of master resilience trainers who will, in turn, be in charge of training others (“train the trainer” kind of approach).

The module on master resilience trainers is already being implemented [3]. It is divided into three components that take place in a 10-day program: preparation, sustainment and enhancement. The preparation component is the main part of this program and focuses of four modules: resilience, building mental toughness, identifying character strengths and strengthening relationships. In every module, different activities are utilized to assure a better understanding of this new information. For instance, each module starts with a brief didactic presentation and is followed by activities (discussions, role plays, exercises, etc.) that allow participants to put into practice newly acquired skills. Sustainment and enhancement components are added as a way to prepare the future use of the new techniques by those trained, allowing them to identify in others difficulties in their resilience skills. The entire Comprehensive Soldier Fitness program is now being empirically tested in outcome trials [15].

The usual training method used to acquire the emotion regulation skills in the Road to Mental Readiness, the Programme d'Entraînement à la Resilience Militaire and the Comprehensive Soldier Fitness programs relies essentially on didactic seminars and short demonstrations in a classroom or a briefing room. Although the teaching method for the Comprehensive Soldier Fitness may differ slightly from the Canadian program [8], [10], SMT is not practiced under stress. To define SMT simply, it is the training in applying any set of techniques aiming to improve how people cope with stress. Coping represents efforts to manage demands, conflicts and pressures that drain, or exceed, a person's resources [16].

As a set of techniques, SMT has been shown effective to control stress and reduce its negative impact (see [17], [18] for reviews). The techniques used in SMT differ greatly from one program to another, ranging from prayer [19] to problem solving [20] and diaphragmatic breathing (referred to as tactical breathing by Grossman & Christensen [21]. Some strategies have received very strong empirical support, especially progressive exposure or “inoculation” to stressful stimuli, relaxation, biofeedback, diaphragmatic and other breathing techniques and cognitive restructuring [17]. Like the acquisition of any new skill, SMT is difficult to master in a stressful situation, and therefore repeated practice is important, usually over several training sessions [22]–[23]. The exact number of sessions or hours of training remain undefined, but attaining a performance-based criterion appears to be optimal (e.g., reaching a pre-defined level of stress control or perceived self-efficacy) and is usually the standard in psychotherapeutic applications [17], [22]. Nevertheless, in mental resilience programs delivered in the military, training as usual does not involve rigorous practice.

Only teaching psychological coping skills during lectures and role plays, no matter how long it may be, can hardly be sufficient to lead to effective use of the techniques, especially in stressful situations [17], [18]. Like any behavioural skills, learning in theory how to use a skill transfers very poorly to actual behaviour change in difficult and challenging situations. How can someone become efficient to manage stress if he or she never practices in a stressful situation? The basic principle for effective learning of a coping skill is first to teach and explain the strategy, second to practice coping skill in a simple situation, and finally to progress to more and more challenging situations [6], [22], [23]. It is important to note however that practicing SMT or emotion management skills requires inducing stress, which may be difficult to recreate in didactic settings or role plays in a briefing room.

In addition to the need of practice, another important challenge in the implementation of SMT and emotion regulation skills in soldiers is the potential resistance to practice and use tools developed to deal with emotion regulation. In a report by the US Department of Defense Mental Health Advisory Team [24], over half of the soldiers surveyed reported that they believed seeking psychological help would lead them to being perceived as weak. Also, almost half of the sample felt that, had they required help for a psychological problem, their unit leader might have treated them differently and members of their unit would have had less confidence in them. Although this data focuses more on seeking treatment than using prevention tools, it fits well with current knowledge in masculine gender role [25]. In accordance with well described and documented male socialization processes [26], [27] and documented implicit masculine standards in the military culture [5], [28]–[30], soldiers may very likely be reluctant to practice and use skills developed for the purpose of regulating emotions.

Immersive technologies such as virtual reality and 3D games provide an interesting solution to the problems of soldiers not buying-in and being non-compliant with the practice of SMT [6], [18], [31]. Bouchard et al. [32] tested different 3D games and immersive technologies and found that games combining the horror and the first-person shooter genres could be useful stressors to practice stress management skills. Since a significant percentage of soldier play videogames [33], this technology may be well accepted in this population. The combination of biofeedback with immersive videogames offers many advantages [34], [35]. First, in a situation of stress, a person's attention is rarely focused on the physiological and psychological effects of stress. Trainees would benefit of being continuously informed of their level of arousal while playing a highly captivating videogame so they could learn to better detect signs of stress and use the appropriate coping strategy. Second, biofeedback in the videogame reinforces the learning process with a positive reward and an increase in perceived self-efficacy when stress management skills are mastered. A third benefit is to allow instructors to tailor the training session to the individual's needs. Another advantage is the possibility to force trainees to master their stress management skills in order to win by reducing the player capacity to play efficiently based on real-time physiological data. For example, as the stress level increases the biofeedback program can make aiming at a target more difficult or progressively reduce the player's field of view. A fifth benefit is to maintain motivation to keep practicing by increasing the difficulty level based on real-time physiological data. As opposed to force the trainees to apply their coping strategies in order to have a fair chance to win, the general difficulty level can be increased as the trainees become efficient in managing stress.

The aim of the study was to test whether practicing stress management skills would increase the efficacy of “training as usual” offered to military personnel. In order to reach this goal, a SMT program called Immersion and Practice of Arousal Control Training (ImPACT) was developed where soldiers: (a) were exposed to stressful situations through immersions in a horror/first-person shooter game, and (b) could learn to master their skills with the use of biofeedback. Based on previous publications documenting the efficacy of SMT [17] and arguing for the need to practice these skills in order to become efficient [18], we hypothesized that when facing an objective stressor, participants who receive the ImPACT program would experience less stress than those who only received usual training.

## Methods

### 1. Participants

**Figure 1:**
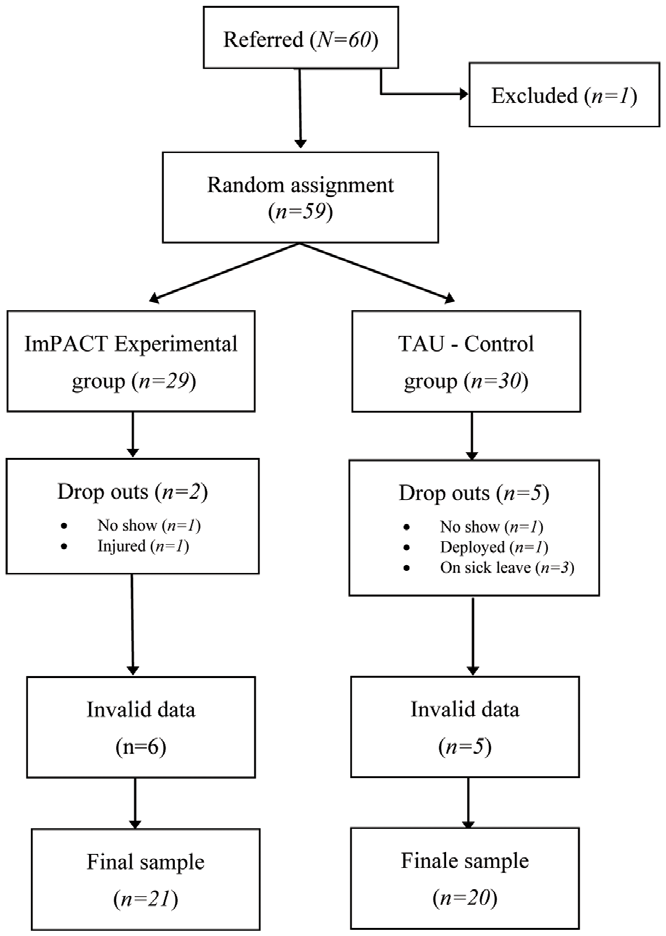
Participants' flowchart.

Soldiers were recruited from a list of volunteers provided by the Valcartier military base of the Canadian Forces. A total of 60 participants were initially recruited, but one was excluded (see selection criteria below) and some others dropped-out or their data were lost during the experiment due to technical problems (see Figure 1 for a flow chart and the Measure section for details). The final sample was composed of 41 male soldiers corresponding to inclusion and exclusion criteria established before the start of the study and approved by the ethics boards of the Canadian Forces and UQO.

The study was open to male and female between 18–60 years old with stereoscopic vision and who had received both: (a) basic stress management program and (b) basic first-aid training. They also had to be physically and mentally fit for duty and, to reduce the risks of cybersickness, not suffering from vestibular problems, recurrent migraines, epilepsy, postural balance problems, cardiac or ocular problems, frequent and intense motion sickness in transports.

Selected participants were randomly assigned to one of two conditions: (a) a “training as usual” control (TAU-Control) condition where no session of supervised practice was offered to participants, or (b) a practice condition (ImPACT) where three daily sessions were offered to practice stress management skills while immersed in a 3D game to induce stress and using biofeedback to inform the participant on their current level of arousal.

The sample of 41 participants can be described as mostly single (68%) young man (mean age of 24.9, SD = 5.55; years of service 4.8, SD = 3.39) with junior military ranks of Private (44%) and Corporal (48%). Sixty-two percent of them had been exposed to combat before. Comparisons between both conditions with Student t tests and chi-squared tests did not reveal any statistically significant difference.

### 2. Procedures

Upon arrival at the Canadian Forces Medical Simulation Centre for pre-screening (Day 1), participants were informed about the study and their right to refuse to participate. They completed an interview with an experimenter who administered the pre-screening survey, performed the Randot stereotest, and were told how to collect cortisol samples. All participants were gathered into groups to receive a 15-minute refresher briefing on SMT. The briefing focused on tactical breathing, a technique they had already learned in their basic usual training. Participants in the TAU-Control condition were told they would come back only on Day 5 for the final part of the study. Participants in the ImPACT condition remained on site and played the game on a 50-inch monoscopic television (TV) until they learned how to navigate in Left 4 Dead.

For participants in the experimental condition, the ImPACT program was delivered on Day 2, 3 and 4 and cortisol samples were taken every day. The training program required participants to wear a system that monitored their heart rate and skin conductance while playing for 30 minutes a modified version of a 3D game. The immersive and biofeedback training was delivered inside two military ambulances located in the medical simulation center. Ambulances were chosen because they allowed the immersion to be conducted in a dark and enclosed environment. Two such ambulance-modified rooms were located about 10 meters from one another and were linked to a cluster of computers allowing participants to work in team (multi-player mode) and a research assistant to supervise all events occurring on the gaming computers and coach's computers. An experimenter, referred to as a coach, sat beside the participant, closed the ambulance door and launched the software. Coaches were experienced retired members of the Canadian Forces. While the game was loading, baseline physiology was recorded during two minutes. During each session, the participant was receiving continuous visual and audio feedback on his stress level. The coach applied the techniques described in the ImPACT trainer's manual. The program is broader than narrow training in tactical breathing. Participants were trained in tactical breathing and also in other associated SMT techniques, such as the recognition of signs of stress, increase in perceived self-efficacy to control stress and biofeedback.

As part of the ImPACT program, tactical breathing was presented to the participant as a tool that can be used in at least three types of contexts: (a) before a stressor creates a peak in stress (i.e., in moments of apprehension), (b) while stressed and performing his duties (i.e., with minimal intrusion on task performance), or (c) while stepping back from the task (i.e., when becoming overwhelmed by stress and stepping back becomes necessary to regain control). All three types of contexts were practiced during the training program. The coaches insisted on first recognizing stress as it increases (discrimination training [36]) and then progressively developing confidence and proficiency in using tactical breathing (self-control [36]). Tactical breathing [21] is a stress management technique where the participant: (a) takes a deep abdominal breath through his nose for a count of four, (b) holds that breath for a count of four, (c) breaths out through his mouth for a count of four, and (d) holds again for a count of four without breathing at all. Session one included a brief recap of the technique and allowed time for guided discovery and familiarization with one's own stress response, getting acquainted with biofeedback while playing with the goal of mastering the skill instead of winning the game, playing the game at a slow to moderate pace and a summary of what was learned in the session. Session two allowed practicing the technique while playing the game at a moderate pace and increasing the pace progressively. It ended with a summary of key learning points. The last session required only one coach who supervised both players while they were applying SMT and playing the game at a more natural pace and guided by biofeedback to autonomously apply tactical breathing. The session ended with a revision of what was learned during the program.

On Day 5, participants came back to the Canadian Forces Medical Simulation Centre for a live first aid simulation. The simulation was intended as a significant stressor that would allow measuring the impact of the program in a situation that is relevant to the work of soldiers in theaters of operations. Participants were tested in pairs at the same time of the day in the simulation, one from each condition, to limit the impact of the chronobiology of cortisol. They could not see each other during the simulation as both simulations were separated by curtain walls. The soldiers strapped on their chest a Polar belt (see Material section), dressed on with their combat gear, took the pre simulation cortisol sample, entered the darkened simulation area and sat for two minutes to record baseline heart rate. They received their mission, which described a situation in Afghanistan, after sunset, with only a full moon providing limited lighting and an Islamic call for prayer being heard loudly in the background. A fellow soldier was caught in an ambush and they had to help him.

After reading the mission, participants stood up in front of a curtain wall hiding the scene until they got the order to proceed. At the exact moment the curtain opened, a very loud explosion occurred from an improvised explosive device hidden in a garbage can and the fellow soldier was severely wounded at the head (blood), chest (open wound with blood), arm (blood) and leg (severely wounded and dismembered prosthetics). Eight medic actors were recruited to perform, one at a time, the wounded fellow soldier. They were randomized across conditions so for each pair of participants, one actor was randomly assigned to the participant in the ImPACT or the TAU-Control condition. The wounded soldier was lying on the floor, covered with blood, begging for help and suffering from severe pain. The very large room was filled with smoke and a second improvised explosive device exploded while the participants were providing first aid medical assistance. After 10 minutes, the simulation ended and participants took the post-simulation cortisol sample and completed the self-efficacy scale.

### 3. Material

The ImPACT program included a software system composed of: (a) a stressful game to be played by the participant, (b) a visual and audio feedback for the participant, (c) a biofeeback recording system to be used by the coach, and (d) a graphical user interface allowing the coach to fine-tune the biofeedback while the session was progressing. The stressful game was a modified version of “Left 4 Dead” (© 2008 Valve, USA; ESRB rating as “Mature”). This horror/first-person shooter game was found in a previous study [32] to be sufficiently stressful and yet appreciated by soldiers as a training stressor. The game was further modified to increase stress level and participant played in team (multi-player mode) against the computer. Although participants had to collaborate to achieve their mission, they could not communicate verbally with each other during the game. Their objective was described as follow: they were part of a team of three - two male soldiers and one civilian female - who were survivors of an apocalypse which created zombies. They had to exit a hideout and reach a rallying point on a farm while hordes of infected zombies were trying to kill them, and without having the defenseless civilian being killed. A sheet describing the mission and how to navigate in the game was positioned near the participant's keyboard. Navigation was performed with keyboard keys, and looking/firing with the mouse. To reduce the impact of learning how to use the controls, participants were allowed on Day 1 to play briefly (on average for 5 minutes) at an unmodified version of Left 4 Dead on a 50-inch TV (different map, no biofeedback). The notion of cooperative play was fostered during the active training with participants by having to cover each other and protect the civilian who was the only one with the power to restore their health. To increase stress, the following modifications (from the default difficulty level) of the game were performed with SourceMod: (a) the number of zombies was increased by 66%, their artificial intelligence by 50%, their resistance by 50% and their rapidity by 80%; (b) the special infected zombies were increased in number (33% more boomers, hunters smokers, 2% more tanks, no witches), in resistance to damages (50% for boomers, 40% for hunters, 40% for smokers and 25% for tanks), and in rapidity (58% for boomers, 66% for hunters, 48% for smokers and 48% for tanks), (c) the delay for spawning new hordes of zombies varied between zero to 60 seconds, (d) the level of damages caused by friendly fire was increased by 3000%, and (e) the civilian medic's behavior was erratic so she could walk in the line of fire at any time and killed.

Feedback on arousal was provided in part with visual feedback. As participant's arousal was increasing, the display of the game on the TV was reduced with a red texture that partially obstructed the field of view, up to a point where only a small oval portion of the center remained visible. The field of view was not totally obstructed in order to avoid masking the action and stirring a sense of loss of control, and yet it interfered sufficiently with the game to require corrective actions from the user. It also illustrated the concept of tunnel vision that is associated with extreme stress [21].The sound of a pumping heart audio feedback complemented the visual feedback by progressively increasing in frequency and loudness. Pre-recorded heart beats started to provide biofeedback before the red texture became visible and continued to increase in pace and volume after the red texture reached its full opacity. This allowed participants to pay attention to heart beat, which is a natural indicator of stress level, and continued providing useful feedback after the visual display had reached its maximum level.

The biofeedback recording system used by the coaches allowed visualizing in real time on a graph the evolution of the participant's heart rate and skin conductance. It used two channels/screens provided by the Biograph Infinity software from Thought Technology. This information was displayed on the coach's laptop computer and could be used to detect problems with the biofeedback device and, at the end of the training session, showed to participants' changes in their physiology.

The graphical user interface provided on the coach's laptop, next the physiology level, allowed fine tuning the biofeedback delivered to the participant. By referring to a 2-minute baseline recorded while loading the game, both physiological parameters were integrated to provide feedback on arousal. The coach could adjust in real time: (a) the sensitivity of the feedback, (b) the relative weight of the heart rate versus the skin conductance, (c) the baseline level in order to maximise the chances participants could benefit from the biofeedback (e.g., if participants reduced their stress level below baseline they could still get useful feedback on their reaction to stressful event occurring in the game), and (d) the difficulty level of the game (e.g., significantly increasing the number, health and rapidity of the enemies; this option was used during the last day of the experiment to make sure participants would not get caught in the fun of playing and forget practicing SMT). At the end of the immersion, the weight and sensitivity settings were saved to be available for the following training sessions.

The graphical user interface interfaced with Biograph Infinity and Left 4 Dead via a Direct X wrapper programmed in C# to create seamless, timely interaction between the programs. By setting a TCP connection from the host to the client running Left 4 Dead, the data was sent over the network to the appropriate computer running Left 4 Dead, and the modifications made to Left 4 Dead would take effect. Based on available literature on biofeedback [37]–[42] and trial and error iterations, arousal level was estimated according to the following equation:
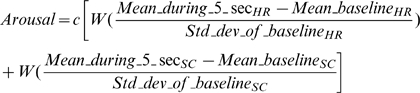



By default, the heart rate and the skin conductance were given an equal relative weight “W” and the sum of both physiological parameters was multiplied by a constant “c” to allow increasing the sensitivity of the feedback.

Based on Bouchard et al. [32] experiment, participants were immersed using 50-inch stereoscopic televisions (Panasonic Viera TC-P50VT25 3D Plasma HDTV, 1080p with a pair of TY-EW3D10 3D glasses) and a Dell laptop computer (XPS L501x, Intel i7 Q740 @ 1.73 GHz, 8 GB of RAM, NVIDIA GeForce GT 435M graphics card, 5.1 Surround Realtek High Definition Audio sound card and running on Windows 7 Ultimate 64-bit). Each provided surround sound through a 5.7 Logitech Z-5500 speaker system. The coach's laptop were more modest computers (one Dell Latitude D630 Intel Core2 Duo T7800 @ 2.60 GHz with 2 GB of RAM with a NVIDIA Quadro NVS 135M graphics card, and one Gateway MA6 Intel Centrino Duo T2300 @ 1.66 GHz with 1 GB of RAM and a standard VGA Adapter on-board graphics card).

In order to test the ability to perceive binocular disparity in distance from static stimuli (stereoscopic vision for depth perception), the participant performed a stereopsis test (Randot™ SO-002, Stereo Optical Company Inc).

### 4. Measures

The main measure of stress in this study was the concentration of salivary cortisol. Free cortisol response collected in the saliva is a reliable measure of stress and, although physical exercise can increase concentration of cortisol in the saliva [43], people regularly trained in physical exercises such as soldiers show an adrenocortical stress and heart rate responses [44]–[47] that are significantly more moderate than non-physically trained people. Heart rate was used as a secondary objective measure of stress. Given the physical efforts inherent to the provision of first aid in combat situations, it was expected that heart rate would peak significantly during the simulation. The maximum heart rate was therefore used as a covariable in the analysis of the cortisol response and mean heart rate was used as an outcome measure and analyzed independently for periods of apprehension and simulation. Two additional measures of the impact of the training program were collected: perceived self-efficacy and medical instructors' “blind” evaluation of participant's performance during the simulation.

Additional measures were also collected to document information relevant to the use of the program. Results on these variables are described to examine how the program was implemented. Socio-demographic information was collected to describe the sample with information on age, gender, military rank, etc.

To assess free cortisol levels, salivary samples were obtained with Salivette™ collection devices (Sarstedt AG & Co). Participants put the cotton sponge swab (without citric acid infusion) directly in their mouth, chewed and rolled the sponge around in their mouth for two minutes and spitted the sponge back into the tube without ever touching the tube. The tube were wrapped in Parafilm® M self-sealing laboratory film and identified by with participant's ID, date and time. Salivary sample were taken at awakening, before and after stressors (i.e., when practicing the ImPACT program and the simulation). The samples were stored and frozen before assaying. The biochemical analysis of free cortisol in saliva was performed with a competitive immunosorbent assay (Salimetrics™ cortisol kit, Salimetrics LLC) using a Sorvall Legend X1R™ centrifuge from Thermo Scientific (Thermo Fisher Scientific Inc) and BioTek ELx800™ microplate reader with GEN 5™ software (BioTek Instruments Inc).

Heart rate was monitored and recorded using a ProComp Infinity™, a wireless-enabling Tele-Infiniti™ Compact Flash T9600 and a wireless EKG receiver from Thought Technology. The electrocardiograph sensor for heart electrical activity was a Polar T31™ wireless transmitter belt from Polar. Skin conductance was also monitored; not as an outcome measure but for biofeedback in the ImPACT program. Skin conductance electrodes were put on the participant's annular and auricular fingers of the hand manipulating the computer mouse. The ProComp Infinity™ sampled the data at 256 Hz. While data was acquired and recorded during the simulation on Day 5, a research assistant double checked that the wireless signal remained good and manually put markers in the series of physiological data at the instant events were starting and ending in order to define four separate blocks of heart rate data: (a) Baseline (2 minutes), (b) Apprehension 1 (when reading the mission and waiting, 3 minutes), (c) Apprehension 2 (facing the curtain wall waiting to waiting to enter the ambush simulation, 20 seconds), and (d) Simulation per se (10 minutes).

Heart rate data was analysed with the Biograph Infinity™ software version 5.1.0 and the Physiology Suite™. The heart rate data of 16 participants was highly erratic during the baseline or the simulation and, to compensate, the built-in algorithm of the Biograph Infinity™ software automatically generated a fixed heart rate (e.g., a flat heart rate response of 170 beats per minutes). In five cases, the original data recorded by the ProComp™ could be salvaged with an, at the time, unreleased version of the Cardio Pro Infinity™ software and guidance from staff at Thought Technology. However, data from 11 participants (see Figure 1) could not be reliably extracted because the data signal was too erratic and noisy.

Perceived self-efficacy [48] to control stress was assessed with a 4-item scale developed for this study. It addressed participant's confidence, on a zero to 100 scale, they could control their stress when experienced at four different intensities. The Cronbach's alpha was .91 when analyzed with 60 soldiers.

The Trainees Evaluation Sheet was completed by professional military medical instructors while soldiers were delivering first aid during the live simulation. It consisted in a list of six essential tasks that had to be performed according to standard training protocol for delivering first aid in combat, otherwise the wounded patient would most likely not survive. It included: (a) assess the scene for safety, (b) assess breathing and correct it if needed, (c) assess blood circulation and correct it if needed, (d) identify appropriate treatment for the chest wound, (e) apply appropriate treatment for the chest wound, (f) assess patient's evolution and adapt to it if necessary. During the simulation, a medical instructor from the simulation center (blind to the participant's condition) remained less than a meter from the participant and continuously assessed the participant's performance in the application of the medical protocol with the wounded soldier. The simulations were video recorded with 16 cameras to allow reviewing the performance so medical instructors could revise their assessment if needed. Medical instructor's assessment were reviewed in a meeting after the experiment to cross-check the ratings. The Trainees Evaluation Sheet was expected to provide behavioural indices of stress. The percentage of participant who performed adequately each behaviour was calculated, as well as the number of participant who did a perfect performance (i.e., zero failure).

The Simulator Sickness Questionnaire [49] was used after each session of the ImPACT program to document that side effects induced by the immersion were not a source of worry for the dissemination of the program. It used 16 different items related to symptoms (headache, eyestrains, nausea, etc.) and raw total scores (i.e., not weighted) were analyzed. The raw (unweighted) total scores were computed and reported [50].

The ImPACT coach checklist was completed by the coaches immediately after each participant's session. It included nine items about elements of the program that had to be addressed with the participant (e.g., reviewed the three contexts where tactical breathing could be applied, helped detect signs of stress, helped apply tactical breathing technique, reinforced the user when he did good) and three items documenting the use of the controls in the graphical user interface (adjust sensitivity, respective weight and value of the baseline).

A 13-item survey investigated how much participants in the ImPACT program appreciated the experience and how useful they consider the program. Only descriptive results were analysed with this survey.

### 5. Statistical Analysis

Parametric inferential statistical analyses (e.g., ANOVA) were performed, except when variables were categorical (e.g., gender, chi-square was then used). Normality of distribution, as measured with the Kolmogorov-Smirnov (values higher than .21, p<.025) and the Shapiro-Wilk (.77, p<.001) statistics, was a problem for the cortisol data only. A log transformation corrected the problem and was therefore used for the analyses. Data on the SSQ were skewed and not normally distributed. Since a Wilcoxon non-parametric test essentially confirmed the results of the repeated ANOVAs and this measure is reported only for descriptive purposes, the parametric analyses are reported.

Cortisol was defined as the principal dependent variable. To control the error rate, a family-wise approach was taken [51], where significance levels were reduced within families of set of variables, which included one set for the physiological variables (cortisol and two analyses for heart rate; α = .05/3 = .017), one set for the subjective self-report variable (perceived self-efficacy; α = .05) and one set of performance variables (medical instructors' observation of 6 behaviors during the simulation and the number of cases with no error, α = .05/7 = .007). No correction was applied for exploratory variables used to document how the program was used. Effect sizes were also calculated (and reported in the results as eta squared and qualitative interpretations of Cohen's *f* [52] for the interaction effects in order to provide a clear impression of the impact of the program, free of cumbersome statistical considerations.

To control the impact of experience in the military, all parametrical inferential comparisons for the efficacy of the program included the number of years in the military as covariable. It allowed generalizing the impact of the program independently from the number of years of duty and experience in combat, in applying first aid and in receiving SMT training seminars. To control for the impact of physical activity generated by providing first aid in an ambush (e.g., having to drag the wounded in a safe location) on cortisol, the maximum heart rate experienced during the simulation was used as a covariable in the analysis of cortisol response.

### 6. Ethics

The present study had been approved by the Defence Research and Development Canada Ethics Committee and the Ethics committee of the Université du Québec en Outaouais. All individual participants in this study gave written informed consent prior to their participation and were free to withdraw from the study without prejudice.

## Results

**Figure 2:**
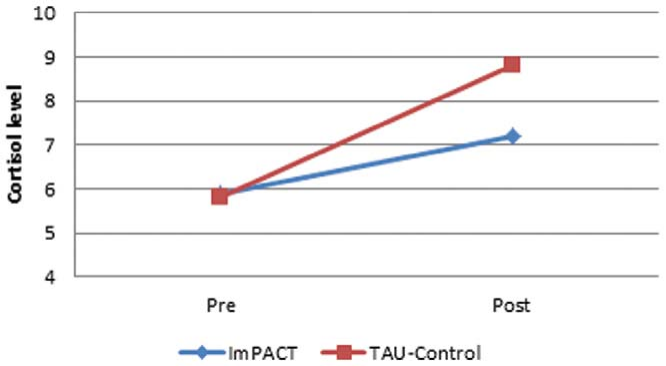
Illustration of a significant difference in stress response as measured with free salivary cortisol (in ug/dL) for participants who received the ImPACT program or training as usual (TAU-Control) before and after a live and stressful simulation.

When comparing efficacy of the ImPACT program on the main measure of stress, there was a significant difference in cortisol response (see Table 1) documenting that the program was effective in better controlling stress than training as usual (see Figure 2) [F_(1,35)_ = 7.31, p<.017, η^2^ = .17, Effect size = very large]. Both the Time [F_(1,35)_ = .28, p = .60, η^2^ = .008, Effect size = small] and Condition main effects [F_(1,35)_ = .0, p = .98, η^2^ = .00, Effect size = trivial] were not significant.

**Table 1:** Cortisol (ug/dL) for participants who received the ImPACT program or Training As Usual before and after a live and stressful simulation.

	Free cortisol levels in ug/dL (standard deviation)	
	ImPACT	TAU-Control
Waking-up	7.79 (4.6)	9.04 (4.36)
Pre simulation	5.93 (2.39)	5.82 (4.47)
Post simulation	7.22 (3.85)	8.8 (4.94)

**Figure 3:**
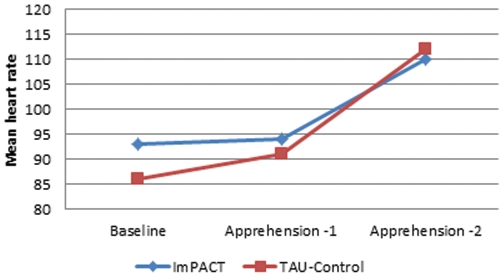
Illustration of the differential increase in heart rate from the baseline to the first apprehension period (when reading the mission) and the second apprehension period (when waiting in front of the curtain wall) phases of a live first aid simulation.

Heart rate was used to corroborate cortisol findings, although the physical efforts inherent to providing first aid in combat situation could blur the heart rate results. The analyses were performed independently for heart rate during the apprehension phases and the actual simulation phases. As shown in Table 2 and Figure 3, the ImPACT program had a significant positive impact on stress levels [F_(2,76)_ = 4.81, p<.017, η^2^ = .11, Effect size = almost large] during the apprehension phases. The Time main affect was significant [F_(2,76)_ = 23.64, p<.017, η^2^ = .38, Effect size = very large] and the Condition main effect was not [F_(1,38)_ = .38, p = .54, η^2^ = .01, Effect size = small]. The contrasts confirmed the superiority of dealing with stress following the ImPACT program compared to TAU-Control when comparing the baseline to the first apprehension phase [F_(1,38)_ = 7.52, p<.017, η^2^ = .17, Effect size = very large] as well as the second apprehension phase [F_(1,38)_ = 7.71, p<.017, η^2^ = .12, Effect size = more than large]. Results were not significant when comparing stress levels during the simulation (see Table 2), as shown by the interaction of the repeated measure ANOVA [F_(1,38)_ = .65, p = .43, ns, η^2^ = .02, Effect size = small]. The Time main effect was significant [F_(1,38)_ = 58.73, p<.025, η^2^ = .61, Effect size = very large], but not the Condition main effect [F_(1,38)_ = .87, p = .36, η^2^ = .02, Effect size = small].

**Table 2:** Average heart rate before and during all phases of the live simulation (Day 5 of the experiment).

	Mean heart rate (standard deviation)	
	ImPACT	TAU-Control
Baseline	93.32 (13.57)	86.26 (9.62)
Apprehension 1	94.08 (11.68)	91.27 (12.80)
Apprehension 2	110.92 (14.12)	112.41 (16.77)
Simulation	132.88 (24.72)	130.86 (21.58)

Ratings of perceived self-efficacy to control stress were quite similar at the start of the study (mean 69.2, SD = 17.52 in the ImPACT condition and 67.21, SD = 23.57 in the TAU-Control condition) but changed at the end (mean 82.69, SD = 6.88 in the ImPACT condition and 59.71, SD = 25.0 in the TAU-Control condition). The repeated measures ANOVA revealed a significant interaction [F_(1,35)_ = 15.8, p<.025, η^2^ = .31, Effect size = very large] and no significant Time and Condition main effects.

Performance during the medical simulation was assessed by medical instructors using the Trainee Evaluation Sheet (see Table 3). Chi-square analyses revealed a significant difference between the conditions on the performance of one critical behaviour (identifying the appropriate treatment to a severe chest wound) and two marginal differences (i.e., they would have been significant if a Bonferroni correction had not been applied) on adapting to the changes in the patient's status and perfect success on the medical test.

**Table 3:** Trainees Evaluation Sheet rated by “blind” military medical instructors during the simulation describing the percentage of participants who followed adequately the official first aid protocol.

	Rated as successfully performed		Chi-Square value (df = 1)
	ImPACT	TAU-Control	
Asses the scene for safety	95%	95%	2.03
Assess respiration and correct in needed	65%	60%	.11
Assess blood circulation and correct if needed	90.0%	89.5%	.003
Identify appropriate treatment - chest wound	58.0%	55.0%	.03
Apply treatment efficiently – chest wound	42.0%	5.0%	7.56*
Adapt to changes in patient's status if needed	68.4%	35.0%	4.36§
Performing without any mistake	45.0%	10.0%	6.14§

Note.

* Values of chi-square p<.007.

§ Values of chi-square p<.05, ns.

The side effects induced by the immersions were small (see Table 4) and did not represent a significant increase from discomfort experienced before the immersion, for session 1 [F_(1,20)_ = 3.07, p = .1, ns, η^2^ = .13, Effect size = almost large], session 2 [F_(1,20)_ = 3.94, p = .06, ns, η^2^ = .16, Effect size = large] and session 3 [F_(1,20)_ = 2.45, p = .13, ns, η^2^ = .11, Effect size = medium].

**Table 4:** Unwanted side effects during each session of the ImPACT program.

	Self-report of simulator sickness		
	Session 1	Session 2	Session 3
Simulator Sickness Questionnaire pre session	2.38 (2.75)	2.14 (3.31)	2.48 (5.21)
Simulator Sickness Questionnaire post session	3.71 (4.78)	3.33 (4.53)	3.67 (4.6)

When systematically reviewing if each component of the program was implemented as intended in the manual, results confirmed the integrity of program delivery. In general, 100% of the content was delivered to each participant at each session, except on two occasions. Only once, during session 1, where a coach forgot to mention the contexts where tactical breathing could be applied and that stress management should be practiced with real stressors. Table 5 describes how often the different options of the graphical user interface were used. Among the stressful events occurring in the game, the hordes of zombies were the most frequent and caused significant increase in heart rate [F_(1,27)_ = 11.01, p<.001] and skin conductance [F_(1,27)_ = 10.83, p<.001] when comparing participants' physiology 10 seconds before and after the beginning of the stressor.

**Table 5:** Use of the available controls at least once during a session by the coaches.

	The option was used at least once		
	Session 1	Session 2	Session 3
Adjust sensitivity	86%	100%	90%
Adjust respective weight	43%	62%	84%
Adjust baseline	43%	53%	70%

After the last day of training, participants completed a survey describing how much they appreciated the program and its components. The descriptive statistics are reported in Table 6. At the end of the survey, participants were invited to express comments about the ImPACT program. Sixty two percent of the participants wrote some comments and all stated they appreciated the program. Two participants suggested allowing communications between the team members would make the game more interesting.

**Table 6:** Descriptive statistics from the survey assessing participant's impression of the ImPACT program on scales ranging from 0 to 10.

	Mean	St. Dev.	Minimum	Maximum
Felt stress during the immersions	6.14	2.02	2	10
Like the immersions	9.05	1.05	7	10
Immersions would be useful to practice SMT	8.38	1.78	5	10
Visual feedback was useful	8.14	1.78	5	10
Presence of the coach was useful to master SMT	8.95	1.61	7	10
How stressful was:	-	-	-	-
Sounds in the game	7.6	2.16	2	10
Quality of the images	7.3	2.34	2	10
Visual feedback of heart rate	8.35	1.81	4	10
Darkness	6.55	2.84	0	10
Playing the game	7.4	2.04	3	10
Playing with a colleague	6.45	3.46	0	10
Effects of surprise	7.8	2.24	3	10
Forewarning effects	7.4	1.85	3	10

## Discussion

Stress management training encompasses a broad range of techniques that are used either very specifically to regulate emotions or as part of broader mental fitness and resilience programs. Acquiring the psychological skills to cope with stress requires practicing under stressful conditions and coaching. The innovation in the current study is not the demonstration that SMT is effective to reduce stress. This has been demonstrated in hundreds of studies (for a review, see [17]), although only a handful of studies have look specifically at coping with acute stress [17]. The originality lies in the demonstration that practice is more effective than training as usual, which rests on formal descriptions of techniques and very brief practice in a meeting room. Another original contribution is that, to practice SMT, we developed the ImPACT program, which essentially consists of three immersions in an interactive 3D environment in order to stress the trainees, provide continuous biofeedback on arousal level, and actively practice tactical breathing with the help of a coach. It was our contention that was these ingredients were lacking in mental resilience packages provided to military personnel [10], [13], [53]. Another issue that deserved to be addressed in current programs was the low motivation, or poor “buy-in” factor, toward emotion regulations. Practicing these techniques that may be considered by soldiers as “too feminine for a real man” [25] or a sign of weakness [5].

In a stressful live simulation providing first aid to a fellow soldier wounded by an improvised explosive device, participants that have been trained with the ImPACT program were significantly less stressed than those who only received the training as usual. This is strongly supported with a statistically significant and fairly large difference in cortisol response corresponding to more than a two-fold increase in those who did not receive the ImPACT training. Average scores of salivary cortisol concentration are hard to interpret in reference to standard values because of its chronobiology and the very wide range of normal values. The current data can be tentatively compared to the cortisol response of physically trained adults undergoing a standardized stressor task called the Trier Social Stress Test (TSST) [44] and our program reduced the stress level by more than a half.

In order to corroborate the cortisol findings and trying to tease out if the program was effective both in times of apprehension and action, heart rate was examined during the simulation. Heart rate of participants who received only the default training increased significantly more while being briefed on their mission than the trained soldiers, as well as while waiting for the action knowing that a crisis was about to happen in a few moments and their performance would be formerly evaluated. Comparisons with results found in other studies suggest that, compared to physically trained men undergoing the TSST, our participant's heart rate was higher in both conditions during the apprehension phase and the simulation phase. Heart rate was also higher than in previous study [32] conducted on a similar sample during a TSST.

The program had a statistically significant impact on medical actions performed by participants during the simulation. Significantly more participants treated adequately the patient's chest wound after having received the ImPACT program. The overall quality of the trainee's performance was much better, with perfect application of the medical protocol in 45% of the participants in the ImPACT condition compared to 10% in those in the TAU condition. This difference was not statistically significant when applying our stringent correction for the number of statistical analyses performed, although the probability was lower than .05. A slightly larger sample, or measuring a few less variables, would have made this difference significant. Interestingly, it appears that differences were more important in behaviours that required more cognitive attention. The program also had a clear positive impact on perceived self-efficacy to control stress. After the simulation, participants trained with the program showed an increase in self-efficacy while those in the control condition showed a decrease. Since self-efficacy is a very strong predictor of behavior change [53], this result suggests that soldiers who received the program are more likely to apply tactical breathing in the future. A survey administered to the participants revealed that those who received the ImPACT program clearly liked it (with a mean score of 9 out of 10, and with 7 as the lowest value). After using it, they considered the program and the role of the coach as useful to practice SMT.

Data gathered during the application of the program per se suggests the immersions did not induce significant negative side effects. Interpretation of this result is still difficult given the skewed distribution of scores and the increase in some side effects likely due to anxiety instead of simulator sickness [50]. All important components of the program were addressed by the coaches, as demonstrated by checklists showing a 95% to 100% adherence to what was expected to be delivered.

Not all results unanimously confirmed the efficacy of the program. The advantage of the program over training as usual was not significant when comparing heart rate during the simulation. The analyses revealed a very large and significant increase in heart rate, without meaningful differences between the conditions. It is suspected that heart rate, which was on average around 130 beats per minutes, may have been high in part because of the physical efforts required during the task. Yet, it remains possible that SMT was either: (a) not practiced in situations that were stressful enough for the participant's mastery of the skills to transfer adequately to very demanding stressors; (b) participants may not have applied SMT at all during the very demanding stressor; or (c) the program was not effective. These alternative hypotheses remain to be demonstrated. If the ImPACT program had been effective only for moderate stressors such as apprehension, and not the rest of the live simulation, the cortisol response would not have been so strongly supportive of the program's efficacy. Given the duration of the live simulation and the high heart rates, it would have most likely blunted the cortisol response and the effect of the program would not have been large and significant. As for being ineffective, this is highly unlikely given the significant difference in salivary cortisol and previous findings from the literature [17], [21].

Further research is needed in at least three areas: (a) test the outcome of the ImPACT program on behaviors observed in other stressful situations, such as on a shooting range, when under enemy fire, dealing with hostile reactions from civilians, etc. [54], (b) investigate if the horror/first-person shooter game component of the program could be replaced by stressful scenarios implemented in an existing military simulation training environment, and (c) test if it is possible to improve the ImPACT program by inducing more stress during the immersion, by adding more sessions, or by adding a session with a live simulated stressor. Testing the usefulness of adding practice with higher levels of stress, probably in a fourth training session, seems warranted. Scientifically, it would help clarifying some of the heart rate results. Clinically, it would make sense and remain consistent with one the principle behind the program, which is that behavioral skills are mastered through practice. Practically, it would clarify whether it is necessary to use more real-life and realistic stressors and to what extent the use of stress management skills generalize to novel settings [55].

The most recent and comprehensive stress management program developed in the US for the military population is deeply rooted in concepts such as resilience, positive psychology and self-regulation. The ImPACT program blends well with this approach since trainees are given the opportunity to practice and master a skill that fosters emotion regulation. It is not explicitly focusing on negative emotions, the prevention of mental disorders or building mental toughness. It could complement psychological resilience programs by offering the opportunity to practice under stress, which is hardly feasible otherwise. The costs are minimal and the buy-in factor is strong. The ImPACT program could also be used to complement training program used during military survival school [56], law enforcement training [57] or any other form of SMT. Taylor et al. [56] provided training in arousal control to US Navy personnel enrolled in survival training and relied on participants recalling a past stressful experience to practice breathing control techniques during two sessions. Their results revealed that despite the known effectiveness of stress management [17], the participants trained with their program did not fared better at coping with a stressful simulation than the control participants who had not received the training. It is quite likely that Taylor et al.'s [56] participants would have benefited from practice with more challenging stressors than souvenirs of past events, from coaching and supervision in the application of their stress management skills, from learning cues that would facilitate remembering to apply arousal control techniques, and from more practice than two 40-minute sessions where the available time is divided into learning four strategies instead of mastering one efficiently.

To conclude, it is doubtful that the classical approach to SMT in the military, which is essentially based on providing information and teaching techniques in a classroom, would be sufficient to result in significant mastery of the techniques. Practice is essential; yet it may be insufficient unless it is performed under stress. Objective information about level of arousal and immediate feedback about the impact of the techniques can greatly improve learning. Soldiers should also be interested by the available learning strategy despite the strong masculine standards found in the military culture [27], [28], [30]. The ImPACT program provides immersions in a stressful game coupled with biofeedback, is attractive to soldiers, and induces enough stress to practice SMT and enough feedback to allow soldiers mastering the technique and increasing their perceived self-efficacy.
